# Influence of CYP2C19 Genotypes on the Occurrence of Adverse Drug Reactions of Voriconazole among Hematological Patients after Allo-HSCT

**DOI:** 10.1007/s12253-017-0264-9

**Published:** 2017-07-06

**Authors:** Beata Sienkiewicz, Donata Urbaniak-Kujda, Jarosław Dybko, Andrzej Dryś, Magdalena Hurkacz, Tomasz Wróbel, Anna Wiela-Hojeńska

**Affiliations:** 1 Department of Clinical Pharmacology, Faculty of Pharmacy, Wrocław Medical University, 211a Borowska St, 50-556 Wrocław, Poland; 2 Department and Clinic of Haematology, Blood Neoplasms, and Bone Marrow Transplantation, Wrocław Medical University, 4 Wybrzeże Pasteura St, 50-367 Wrocław, Poland; 3 Department of Physical Chemistry, Wrocław Medical University, 211a Borowska St, 50-556 Wrocław, Poland

**Keywords:** Adverse drug reactions, Voriconazole, CYP2C19, Genotyping, Hematology

## Abstract

The aim of this study was to determine the influence of different CYP2C19 genotypes on selected liver function parameters, and ADR occurrence during VCZ prophylaxis in adult patients after allo-HSCT (allogeneic hematopoietic stem cell transplantation). CYP2C19 mutations were determined in a cohort of 30 adults using PCR-RFLP methods established by Sim et al. and Goldstein and Blaisdell. The patients’ protocol included biometrical and biochemical data, information on the underlying disease, chemotherapy, molds infections occurring during VCZ treatment, adverse drug reactions typical for the use of voriconazole, and probable drug - drug interactions. The observation and reporting of ADR took place from the −1 until the +20th day of VCZ therapy. For statistical analysis the χ2 test was used (*p* < 0.05). Among the examined patients 23 suffered from at least one side effect during VCZ therapy. Most frequent ADR were gastrointestinal disturbances (*n* = 15), nervous system (*n* = 11) and skin (*n* = 7) disorders. Patients with at least one loss of function allele (*2) were more likely to experience adverse drug reactions than those, with different genotypes. Due to the limited number of patients the result could not be proven with a statistical significance. Previous determination of CYP2C19 genotype may be a useful tool for prevention of adverse drug reactions during VCZ prophylaxis among patients after allo-HSCT.

## Introduction

Antifungal prophylaxis is crucial for the success of each hematologic treatment. Especially for patients classified as those at highest risk of developing invasive fungal infections (IFIs). These are patients receiving intensive chemotherapy for acute myeloid leukemia and myelodysplastic syndrome, as well as corticosteroid therapy for graft-versus-host disease (GVHD) after allogeneic hematopoietic stem cell transplantation (allo-HSCT) [1].

In this group of patients, IFIs are mainly caused by *Aspergillus spp.* and *Candida spp.* The mortality rates are 56% for invasive aspergillosis and approximately 10–25% for candidiasis, what supports the necessity for appropriate antifungal prophylaxis [1–3].

One of the new generation triazol antifungal agents with broad-spectrum activity is voriconazole (VCZ). It is indicated for treatment of invasive aspergillosis, candidemia in non-neutropenic patients, fluconazole-resistant serious invasive *Candida spp.* infections and serious fungal infections caused by *Scedosporium spp.* and *Fusarium spp.* VCZ can also be an alternative for posaconazole in the prophylaxis of high risk patients in hematological units [1, 4]. The agent has saturable nonlinear pharmacokinetics, which properties are mainly influenced by food intake, inter–individual variability and drug-drug interactions. The drug is almost completely absorbed when administered under fasted conditions. The liver metabolism is primarily conducted by the hepatic cytochrome P450 CYP2C19 isoenzyme, and to a lesser extent by CYP3A4 and CYP2C9. Significant genetic polymorphisms in the CYP2C19 gene encoding for the CYP2C19 enzyme may result phenotypically in rapid or slow metabolism of voriconazole, possibly resulting in approximately 30–50% variation of plasma concentrations [5, 6]. This is why many authors postulate the need for CYP2C19 genotyping as a part of therapeutic drug monitoring of VCZ concentrations to avoid adverse drug reactions (ADRs) [7].

Voriconazole may lead to neurological (agitation, dizziness, confusion, anxiety, tremor, auditory and visual hallucinations), respiratory, thoracic and mediastinal, gastrointestinal, hepatobiliary (significant transaminitis) and skin disorders (rash, pruritus, photosensitivity). Pyrexia is another common adverse drug reaction [4]. Dose-related visual disturbances (blurred vision, photophobia, altered visual and color perception) occur in 22–45% of patients. Cardiovascular events (QT prolongation and *torsade de pointes*) have been reported rarely, especially with other risk factors e.g. cardiomyopathy or pro-arrhythmic medications [8]. Drug-drug interactions during hematologic treatment are also important factors leading to changes in VCZ pharmacokinetic parameters. The co-administration of e.g. cyclosporine A, phenytoin or fluconazole was reported to cause side effects due to variabilities in pharmacokinetic properties of the drug [9–11]. Other important drug-drug interaction during VCZ treatment are presented in Table 1.

**Table 1:** Drugs contraindicated during VCZ treatment [4, 10, 12–14]

Drug	Effect of co-medication	Recommendation
Astemizol, cisapride, pimozide, quinidine, terfenadine	Prolongation of QTc	Contraindicated
Carbamazepine, long acting barbiturates	Lower VCZ concentrations	
Efavirenz (doses 400 mg or higher)	Lower VCZ concentrations	
Ergot alkaloids	Possible egotism	
Fluconazole	Higher VCZ concentrations	
Fosamprenavir boosted with low-dose ritonavir	Lower VCZ concentrations	
Lopinavir boosted with high-dose ritonavir	Lower VCZ concentrations	
Rifampicin	Lower VCZ concentrations	
St. John’s Wort	Lower VCZ concentrations	
Sirolimus	Higher sirolimus concentrations	
Simvastatin	Higher simvastatin concentrations	

In a previously published article, we describe 4 patients with CYP2C19*2/*17 genotype experiencing more often side effects of voriconazole then others treated with the same protocols [15]. The aim of this study was to determine the influence of different CYP2C19 genotypes on selected liver function parameters, and ADR occurrence during voriconazole treatment in a larger number of adult patients after allo-HSCT. We also performed a concomitant drug–drug interaction analysis to indicate other factors potentially causing side effects.

## Materials and Methods

In the conducted study, carried out with the permission of the Bioethics Committee of Wrocław Medical University, a cohort of 30 patients, 11 women and 19 men, was examined. An observation and reporting of ADR took place from the −1 day before VCZ administration till the +20th day of treatment.

### DNA Isolation and Genotyping

The material used for genetic testing was whole blood, drawn on the anticoagulant ethylenediaminetetraacetic acid (EDTA). We isolated DNA using the QIAamp® DNA Blood Mini Kit, according to the manufacturer’s instruction in a laminar flow cabinet. Two modified PCR-RFLP methods, previously adapted to the requirements of the Pharmacogenetics and Pharmacogenomics Laboratory of the Department of Clinical Pharmacology, for CYP2C19 allele 1, 2 and 17 determination by Goldstein and Blaisdell and Sim et al. were used [16, 17]. Most important information on the methods are presented in Table 2.

**Table 2:** Methodology used for CYP2C19*2 and *17 determination [16, 17]

CYP2C19 allele	Primer sequences	PCR conditions	PCR product size (base pair)	Restriction enzyme	Restriction fragment size (base pair)		Marker
					Wild type	Variant allele	
*17	5′-AATAAAGATGACCTTGATCTGG-3′ 5′-GTCTCCTGAAGTGTCTGTAC-3’	2 min at 94 °C; 35 cycles of 30 s. at 52 °C, 30 s. at 72 °C, 30 s. at 94 °C; and 7 min. at 72 °C	500	MnII	280, 224	500	DNA M1 Marker
*2	5’-CAGAGCTTGGCATATTGTATC-3′ 5′-GTAAACACACAACTAGTCAATG-3’	5 min at 94 °C; 37 cycles of 30 s. at 54 °C, 30 s. at 72 °C, 30 s. at 94 °C; and 5 min. at 72 °C	321	Sma I	212, 109	321	DNA M1 Marker

### Patients’ Observation Protocol

Considering literature and summaries of product characteristics a patient’s protocol with most important biometrical and biochemical data was evaluated. The protocol also included information on the underlying disease, chemotherapy, molds infections occurring during VCZ treatment, adverse drug reactions typical for the use of voriconazole, and probable drug - drug interactions.

### Treatment Protocol

Twenty-three patients were treated with reduced-intensity conditioning (RIC) and 7, suffering from ALL, with myeloablative conditioning (MAC). The treatment protocols were described previously by Sienkiewicz et al. [15].

### Statistical Analysis

Statistical analysis was performed with the STATISTICA statistical software (STATISTICA 12, StatSoft, Tulsa, Okl.). *P* values less than 0.05 were considered statistically significant. To determine the influence of variables such as BMI and genotype on the occurrence and frequencies of ADR the χ2 test was used.

## Results

In the examined cohort the median age was 52 years and the median BMI was 26. Ten patients were overweight (with BMI values over 25). All of the recruited patients underwent hematopoietic stem cell transplantation at the Department and Clinic of Haematology, Blood Neoplasms, and Bone Marrow Transplantation of Wrocław Medical University between 2015 and 2016 due to; acute myeloid leukemia (AML) 11 cases, acute lymphoblastic leukemia (ALL) 7 cases, myelodysplastic syndrome (MDS) 6 cases, aplastic anemia (AA) 2 cases, multiple myeloma (MM) 2 cases, myeloproliferative syndrome (MPS) 1 case, and lymphoma in 1 case.

**Fig. 1:**
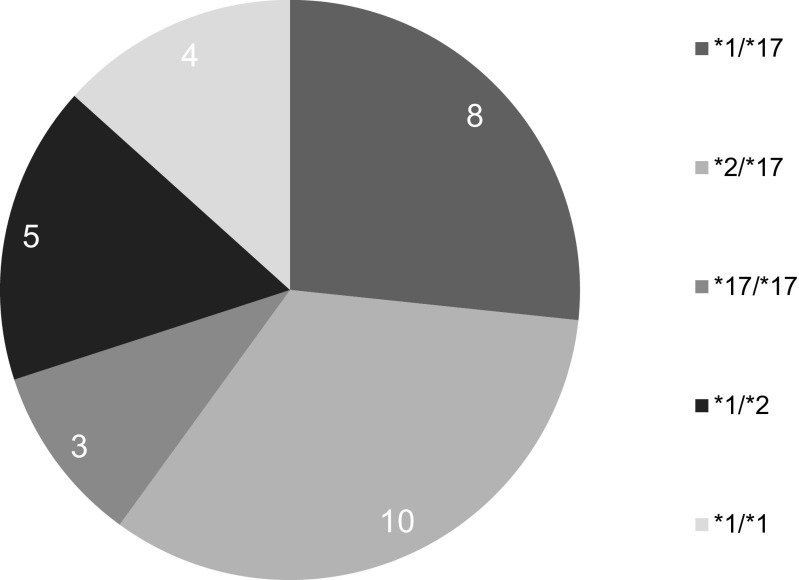
CYP2C19 genotypes among the examined patients

The CYP2C19 genotypes found by performed genetic testing are presented in Fig. 1.

**Fig. 2:**
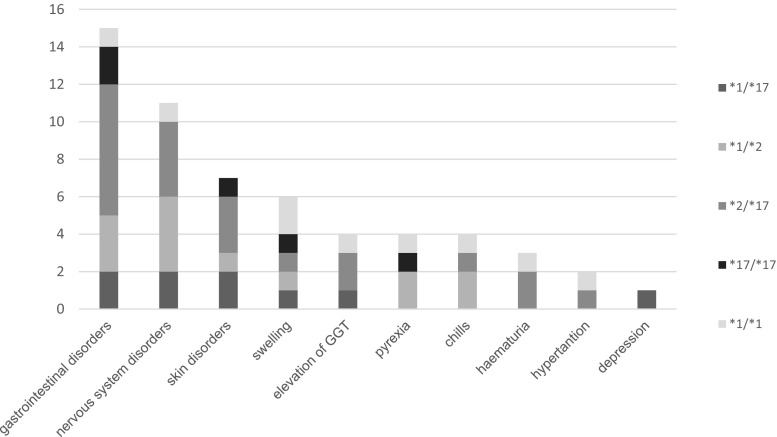
ADR in relation to CYP2C19 among the examined patients cohort

VCZ prophylaxis failed in two cases, where an invasive pulmonary aspergillosis occurred. Twenty-three patients suffered from at least one side effect during therapy. The experienced adverse drug reactions among the patients’ cohort in relation to CYP2C19 genotypes are shown in Fig. 2. Most frequent ADR were gastrointestinal disturbances (*n* = 15), nervous system (*n* = 11) and skin (*n* = 7) disorders.

Patients demonstrating the CYP2C19*1/*17 genotype suffered mainly from skin (rash, erythema), nervous system (headache, vertigo), and gastrointestinal disorders (nausea, gastritis). Patients with CYP2C19*1/*2 genotype experienced nervous system (headache, vertigo) and gastrointestinal disorders (constipation, nausea), CYP2C19*2/*17 patients suffered from gastrointestinal (nausea, vomiting), nervous system (headache, vertigo), skin disorders (erythema, rash), haematuria and elevated GGT values. Patients with CYP2C19*17/*17 genotype experienced vomiting and skin disorders whereas *wild type* genotype was connected with swelling (face and peripheral oedema). According to the presented results, patients with at least one loss of function allele (*2) experienced adverse drug reactions more often than those with different genotypes. Due to the limited number of patients the result could not be proven with a statistical significance. As for published case reports suggesting an impact of BMI on ADR we also tried to determine if there is such an influence among the examined cohort of patients [18, 19]. No relationship between BMI and frequency of side effects was found. The presented complications were temporary and had no impact on the dosage regimen nor the conducted pharmacotherapy. After convalescence, the patients were discharged from hospital.

## Discussion

Thirty patients were recruited for our study. The most frequent adverse effects were gastrointestinal disturbances, skin disorders, headache, swelling, liver abnormalities (elevated GGT), pyrexia, chills, haematuria, hypertension and depression respectively. To our knowledge there are few studies on the topic of adverse drug reactions caused by voriconazole in the group of adult HSCT-patients. In a study by Brüggemann et al. visual and gastrointestinal disorders appeared most often [20]. Kim et al. and Chu et al. reported liver function abnormalities, gastrointestinal, renal, skin and visual disorders, appearing with different frequencies [21, 22].

In our cohort ten patients were overweight, no correlation with an increased number of adverse drug reactions among this group could be proven. Our findings are consistent with those from Koselke et al. who also did not observe toxicity differences in a group of 21 overweight patients, although a correlation between strongly obese patients and sub-therapeutic concentrations of voriconazole (used in standard doses) was found [23].

In the conducted study no statistical correlation between CYP2C19 genotype and the frequencies of adverse drug reactions, but a tendency of patients with loss of function allele to experience side effects more often was found. As CYP2C19 mutations are connected with lower (allele*17) or higher (allele*2) VCZ concentrations after standard dosing, an impact on the toxicity of the drug is plausible. Pascual et al. found a correlation between high VCZ concentrations and neurological disorders [24]. In a review Dolton et al. also identified a relationship between voriconazole concentrations hepatobiliary and visual disorders [25]. Our different findings can be due to a limited number of patients, similarly to the study conducted by Kim et al., where no correlation between CYP2C19 genotypes and VCZ concentrations was found [21]. Another factor having an impact on our results could be the concomitant use of cyclosporine A and methotrexate. Voriconazole is an P-gp inhibitor leading to elevated cyclosporine A concentrations, and its ADR [4]. Methotrexate use may also cause adverse drug reactions such as depression, headache, pneumonia and haematuria [26].

## Conclusion

Adult patients after allo-HSCT, demonstrating minimum one loss of function allele of CYP2C19 isoenzyme, are more likely to experience adverse drug reactions during VCZ prophylaxis. Previous determination of CYP2C19 genotype may be a useful tool for prevention of adverse drug reactions during VCZ prophylaxis among hematologic patients after allo-HSCT.

There is a need for more investigation on the topic of voriconazoles’ ADR in relation to CYP2C19 genotype, which could help to identify patients potentially more likely to experience side effects.
